# Urine and Serum Exosomes as Novel Biomarkers in Detection of Bladder Cancer

**DOI:** 10.31557/APJCP.2019.20.7.2219

**Published:** 2019

**Authors:** Fathia Elsharkawi, Mahmoud Elsabah, Marwa Shabayek, Hussein Khaled

**Affiliations:** 1 *Department of Biochemistry and Molecular Biology, Faculty of Pharmacy, Helwan University, *; 2 *Department of Biochemistry, Faculty of Pharmaceutical Sciences and Pharmaceutical Industries, Future University, *; 3 *National Cancer Institute, Cairo University, Cairo, Egypt. *

**Keywords:** Bladder cancer- serum- urine- exosomes

## Abstract

**Background:**

The gold standard for initial clinical diagnosis of bladder cancer involves cystoscopic examination of bladder and histological evaluation of tissues. There is a critical need to identify non-invasive and sensitive biomarkers. Early detection is essential challenge in diagnosis and surveillance of bladder carcinoma. Exosomes are nano- sized vesicles present in many biological fluids and have significant role in cancer. Thus, quantification of exosomes in different stages of bladder cancer may be of critical concern for clinical diagnosis and prognosis.

**Methods:**

Tumor derived exosomes levels in urine and serum samples of 70 bladder cancer Egyptian patients from stages T0-T3 and 12 healthy control people were measured using ELISA technique.

**Results:**

When compared to health subjects, exosomes levels in bladder cancer patients were increased in urine and serum samples at different stages of the disease. A gradual increase in tumor derived exosomes in serum (1.21, 3.31, 4.71, 6.47µg/ml) and urine (1.59, 2.84, 4.75, 6.67µg/ml) was observed comparative to invasiveness of tumor (T0-T3). Serum was more specific (100%) sample for detection of exosomes in bladder cancer.

**Conclusion:**

our findings suggest that tumor derived exosomes may offer a convenient tool for early diagnosis and monitoring of bladder cancer.

## Introduction

In Egypt, bladder cancer accounts for approximately 19% of the total incidence of cancers, and it is one of the common type of cancer in men and second most prevalent in females (Antoni et al., 2017). Bladder cancer prevalence has been increased by a rate not comparable with progression speed in biomarkers discovery (Franzen et al., 2014). 

The gold standard for initial clinical diagnosis of bladder cancer involves cystoscopic examination of bladder and histological evaluation of tissue obtained by transurethral resection (TUR) (Babjuk et al., 2013). Therefore, there is a critical need to identify novel biomarkers and target therapies to detect and treat bladder cancer (Jemal et al., 2010).

Exosomes and microvesicles (apoptotic bodies and ectosomes) are extracellular vesicles (EVs) secreted by variety of human cells and have a significant role in cellular communications as transport of nucleic acids and proteins. They have been emerged as novel mediators of tumor progression over recent decades (Gangoda et al., 2015). Also they have ability to protect their components from degradation by enzymes present in extracellular space (Katsuda et al., 2014). Exosomes are nano-sized vesicles with diameter 40–100 nm, released upon fusion of multivesicular bodies with plasma membranes or by direct budding from plasma membrane (Willms et al., 2016). They are round or a cup shaped, present in most biological fluids such as blood, urine, ascites, amniotic fluid, bronchoalveolar lavage and tears (Yu et al., 2015). Exosomes secreted either constitutive after fusion of late endocytic compartments with plasma membrane after activation of Ca^2+^ dependent manner or activation of Rab-GTPases (Blott and Griffiths, 2002), or secreted inducible by variety of signaling pathways such as stimulation of Wnt pathway (Ekström et al., 2014). In addition to that acidic microenvironment around tumors stimulates release of exosomes (Steinbichler et al., 2017). Exosomes play a role in adaptive immune responses through initiating MHC (Major histocompatibility complex) – peptide complexes to specific T cells (Bobrie et al., 2011). Exosomes derived from tumors could change phenotype of normal cells by introducing proteins and RNAs developing metastasis, carcinogenesis, proliferation and angiogenesis (Vaderet al., 2014). Moreover, they induce immune-suppression, and preparation of pre-metastatic niches in secondary organs (Ramteke et al., 2015).

Exosomes are cytoplasmic vesicles enclosed in a lipid bilayer (Janas et al., 2015), contain all types of biomolecules like proteins, carbohydrates, lipids, and nucleic acids (Chaharet al., 2015). Most plentiful proteins in exosomes are protein kinases, G proteins, heat shock proteins (HSP70, HSP90), MHC class I and II and tetraspanins including CD9, CD63, CD81 and CD82 (Mathivanan et al., 2010). In addition to protein constituents exosomes are also composed of variety of lipids and lipid-raft-associated proteins originating either at plasma membrane or from endosome compartments including cholesterol, sphinogmyelin, and flotillins (Greening et al., 2015). Exosomes were diagnosed in ovarian cancer (Chen et al., 2017)where they facilitate ovarian cancer invasion (Nakamura et al., 2016) and in breast cancer (Xiang et al., 2009).

Early detection of urological cancers is pivotal for successful treatment and management. The present study evaluates quantification of tumor derived exosomes as non-invasive biomarkers for diagnosis and prognosis of bladder cancer using ELISA technique specific for exosomes surface protein (CD9) (Kowal et al., 2016). As many reviews have been reported that urine sample is most suitable biological sample to determine kidney, bladder and prostate disorders (Royo et al., 2016), so the aim of the present study was measuring and comparing tumor derived exosomes levels in urine and serum samples to find out the most realistic biological sample for diagnosis and prognosis of bladder cancer.

## Materials and Methods

A prospective analysis was performed on 70 bladder cancer Egyptian patients who have attended at cystoscopic unit of National Cancer Institute, Cairo University, and 12 healthy people with no previous history of any urological disorders as a control group. All subjects signed an informed written consent for participation and the study was approved by the research ethics committee for experimental and clinical studies at faculty of pharmacy, Future University, Cairo, Egypt (No. of protocol: REC-FPSPI-2/17). Patients’ demographic data and medical history were obtained from information and statistics department of National Cancer Institute. Tumor staging and grading (T0-T3) were diagnosed according to tumor, necrosis, and metastasis (TNM), The 2002 TNM classification had approved by (UICC) Union International Against Cancer (Sobin, Gospodarowicz et al. 2011). Patients were divided according to their stages into 4 groups from stage T0:T3 Table (1), however, low incidence of bladder cancer stage 4 (T4) owing to low survival rate (Breyer et al., 2017).

*Samples collection and preparation*

Urine and blood samples were collected in sterile containers and vacutainer tubes respectively in the morning. Samples were taken at the time of diagnosis before receiving any treatment.

*Preparation of urine samples*

Collected urine samples were centrifuged in sterile Wasserman tubes (5-15ml) within one hour of collection at 17,000×g for 20 minutes at room temperature (RT) and clear supernatants were filtered using 0.45 µm filters under laminar flow containing HEPA filter (Alvarez et al., 2012).

*Preparation of serum samples*

Serum samples were prepared by 3 cooling centrifugation processes (4°C) at three differential speeds (300×g, 1,200×g and 10,000×g) for 10,20 and 30 minutes respectively to eliminate red blood cells and cellular debris (Li et al., 2015).

Both filtrated urine and serum samples were divided into aliquots and stored at -80°C until used.

*Reagents and chemical used*

ExoQuantTM overall exosome capture and quantification assay kit for determination of urinary and serum exosomes levels provided by BioVision, Inc.155 South Milpitas Blvd. California 95035 with catalog numbers K1202 and K1203.

**Table 1:** Classification of Bladder Cancer Patients

Groups	Sample size (N)
Control	12
Group 1 (T0)	12 (17 %)
Group 2 (T1)	14 (20%)
Group 3 (T2)	24 (34.5%)
Group 4 (T3)	20 (28.5%)

**Table 2:** Urinary Levels of Tumor Derived Exosomes in Bladder Cancer Patients and Control Group

Groups	Sample size	Mean ±SEM
Control	12	0.59 ±0.03
Group 1(T0)	12	1.59* ±0.12
Group2 (T1)	14	2.84* ±0.07
Group 3 (T2)	24	4.75* ±0.04
Group 4 (T3)	20	6.67* ±0.06

The values were expressed as mean ± standard error mean.

* P < 0.001

**Table 3:** Serum Levels of Tumor Derived Exosomes in Bladder Cancer Patients and Control Group

Groups	Sample size	Mean ±SEM
Control	12	0.57± 0.05
Group 1(T0)	12	1.21*± 0.02
Group2 (T1)	14	3.31*± 0.05
Group 3 (T2)	24	4.71*± 0.08
Group 4 (T3)	20	6.47*± 0.3

The values were expressed as mean±standard error mean.

* P < 0.001

**Table 4:** Tumor Derived Exosomes Levels in Urine and Serum of Bladder Cancer Patients

Groups	Mean tumor derived exosomes levels in urine (µg/ml)	Mean tumor derived exosomes levels in serum (µg/ml)
Control	0.59	0.57
Group 1(T_0_)	1.59	1.21
Group 2 (T_1_)	2.84	3.31
Group 3 (T_2_)	4.75	4.71
Group 4 (T_3_)	6.67	6.47

**Figure 1:**
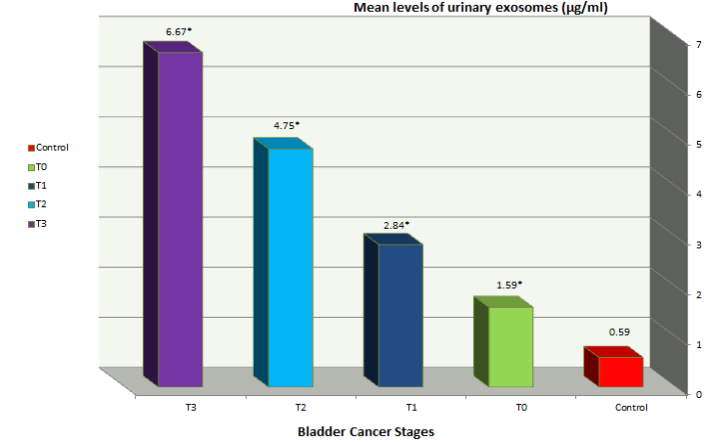
Mean Urinary Levels of Tumor Derived Exosomes in Bladder Cancer Patients and Control Group (µg/ml). *P˂0.001. Mean urinary level of tumor derived exosomes is increasing approximately by steady manner from group 1 to 4 of bladder cancer groups

**Figure 2:**
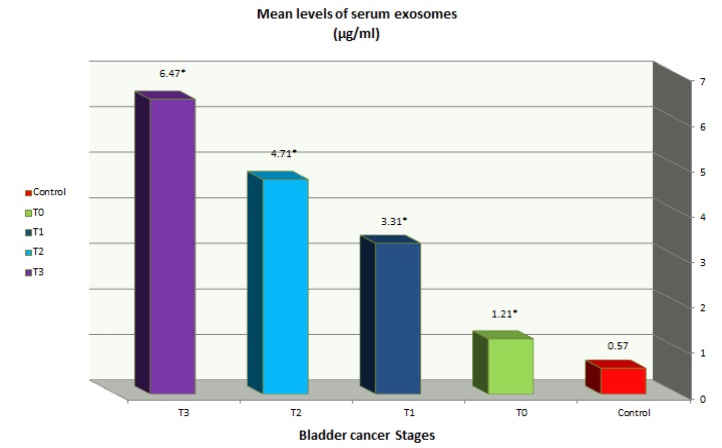
Mean Serum Levels of Tumor Derived Exosomes in Bladder Cancer and Control Groups (µg/ml). *P ˂ 0.001. Mean serum level of tumor derived exosomes is increased gradually from superficial stage (group 1) to invasive stage (group 4), therefore emphasizes on significance of tumor derived exosomes in evaluation and monitoring bladder cancer

**Figure 3:**
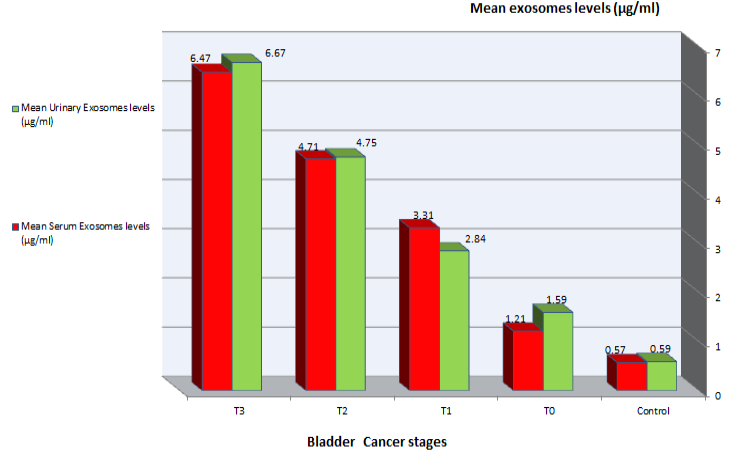
Mean Tumor Derived Exosomes Levels of Urine and Serum at Different Bladder Cancer Stages

**Figure 4:**
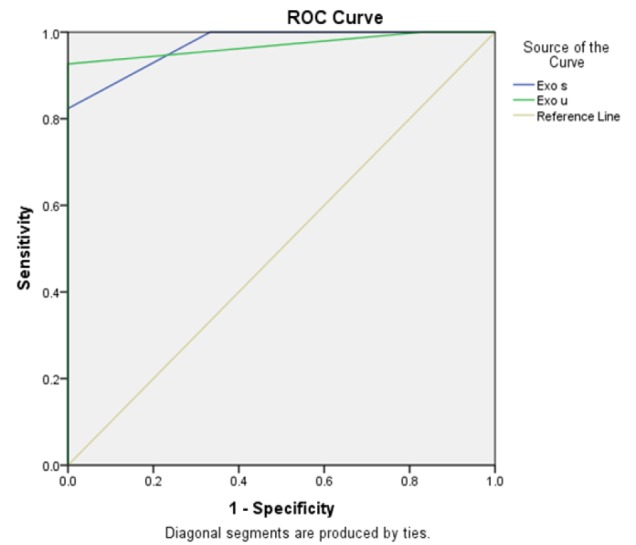
Receiver Operating Characteristic Curve for Urine and Serum Samples. Urine and serum tumor derived exosomes were specific for diagnosis of bladder cancer with 83.3% and 100% specificity and 92.6%& 82.4% sensitivity respectively. Area under curve of urinary tumor derived exosomes was 96.9 and tumor derived serum exosomes was 97.1

*Methods*

Determination of urinary and serum tumor derived exosomes levels using ELISA technique according to manufacturer’s instructions.

The assay is a double sandwich enzyme linked immunoassay for quantitative and qualitative analysis of exosomes. Microplates are pre-coated with exosome capture α-CD9 antibodies. Quantification and characterization of exosomal proteins is performed using appropriate detection antibodies against exosome associated antigens for specific exosomes. Lyophilized exosome standards for assay calibration provided with kit allow the quantification of unknown sample by a standard calibration curve.

According to manufacturer’s instructions 100μl of samples were added to bind exosomes after incubation and washings with washing buffer. 100μl of both mouse anti-human exosome antibody and of rabbit anti-mouse IgG HRP-conjugated secondary antibody solution (for urine) and streptavidin-HRP antibody solution (for serum) were added. Absorbance read at 450 nm within 10 minutes. 

*Statistical analysis*

The SPSS software system for Windows (version 20; SPSS, Chicago, IL, USA) was utilized for statistical analysis. Nonparametric ROC curves generated from data to measure diagnostic accuracy of the measured parameters. Data was presented as mean value ±SEM. 

Differences between the groups were considered significant at P < 0.001

## Results

*Urinary tumor derived exosomes levels*

Statistically significant differences have been observed between urinary exosomes levels of healthy control group (0.59 µg/ml) and all bladder cancer patients (3.96 µg/ml).

There was a gradual increase in exosomes levels between patients’ groups according to severity and invasion of tumor. Levels of exosomes were (1.59 µg /ml, 2.84 µg/ml, 4.75 µg /ml and 6.67µg /ml) in groups 1,2,3 and 4 respectively Table (2), Figure (1).

*Serum tumor derived exosomes levels:*

Statistically significant difference was demonstrated between serum exosomes levels of control group (0.57 µg/ml) and all patients’ groups (3.93 µg/ml).

Table (3) and Figure (2) showed that exosomes levels in each stage of disease were increased relative to control, on the other hand, this increase was associated with tumor invasion since exosomes levels increased by two, six, eight and eleven folds in groups 1to 4 (stages T0-T3 ) respectively .

*III- Tumor derived exosomes levels in urine and serum*

Table (4) and figure (3) showed a comparison of exosomes levels in urine and serum samples with control group, it was found that exosomes levels in both samples were higher than control group and there was a correlation between exosomes levels elevation and invasiveness of bladder cancer. 

*VI- Sensitivity and specificity*

From the created Roc curve, it was observed that specificity of measuring exosomes was 100% in serum and 83.3% in urine while sensitivity was 92.6% in urine and 82.4% in serum samples (Figure 4).

## Discussion

In the current study tumor derived exosomes were utilized in diagnosis and monitoring superficial and invasive stages of bladder cancer. Exosomes as biomarker characterized by high stability, specificity and sensitivity in comparison with other biomarkers (Zhang et al., 2016). The most common methods used for isolation and quantification of exosomes involve ultracentrifugation, filtration and precipitation. The majority of these protocols are still under modification, lengthy (6–8 hrs) with limited efficiency (Cantin et al., 2008, Momen-Heravi et al., 2013). 

The current study relied on detection of CD9 transmembrane protein as biomarker for identifying of exosomes using ELISA technique which provides specific capture and recovery of pure and integral exosomes from different body fluid (Zarovni et al., 2015). 

Some studies used serum exosomes as promising biomarkers for diagnosis of many diseases such as acute ischemic stroke(Ji et al., 2016), renal cell carcinoma (Zhang et al., 2018) and in metastatic and non-metastatic non-small-cell lung cancers (Wang et al., 2018); while plasma exosomes were detected in other diseases as atherosclerotic vascular disease, esophageal squamous cell carcinoma (Matsumoto et al., 2016) and acute myeloid leukemia (Fayyad-Kazan et al., 2013). Most literatures prefer serum to isolate exosomes (Li et al., 2017), since plasma is a complex biological fluid and exosomes isolation process is complicated due to viscosity and density issues(Momen-Heravi et al., 2012), in addition to the presence of more than 90% of total proteins in plasma (Good et al., 2007). Therefore, plasma preparation for efficient exosomes quantification is complex, lengthy process and requiring protocol modification(Caby et al., 2005). 

The current study utilized fresh first morning urine (almost fixed time of sampling) which provides the least variability in protein concentration and centrifugation process was performed within 20 to 30 minutes of collection to minimize contamination of the urine and other handling processes were done under aseptic conditions (Thongboonkerd, 2007). 

It was reported that the level of circulating exosomes in tumor associated individuals is much higher than non-malignant and increases with tumor progression (Toth et al., 2008) and that phenomenon was reported in the current study since urinary and serum cancer cell derived exosomes were correlated with bladder cancer progression in different stages.

It was reported that exosomes convey proangiogenic peptide and miRNA to microvascular endothelial cells to promote tumor angiogenesis (Zhang et al., 2014), in addition to their essential role in facilitating tumor invasion and metastasis (Franzen et al., 2015). On the other hand Franzen et al., (2015) reported that tumor derived exosomes from bladder cancer patients increasing causing urothelial cells to undergo EMT (epithelial mesenchymal transition), and increased migration and invasion (Wee et al., 2018). 

Riches et al., (2016) reported that urinary exosome concentrations in recurrent patients presenting for transurethral resection of bladder tumor were significantly increased than other patients presenting for cystoscopic check with no recurrence, Riches et al., (2016) and that matched with the present study results since tumor derived exosomes levels in urine and serum were increased in association with the invasiveness of tumor and demonstrating beneficial utility of exosomes in prognosis and follow up of bladder cancer patients.

In the present study sensitivity of tumor derived exosomes was higher in urine than serum and that was matched with many literatures, which reported that urine is most suitable biological sample and has a potential to determine subjects with kidney, bladder and prostate disorders (Royo et al., 2016).

Although tumor derived exosomes levels in serum and urine were approximately close in the current study, serum was more specific whereas urine was more sensitive and this outcome may be attributed to high stability of exosomes in biological fluids especially blood over period of time (Kalra et al., 2013). 

In conclusion, our findings suggest that tumor derived exosomes may offer a convenient tool for diagnosis, early detecting or monitoring of bladder cancer. Urine and serum exosomes are recommended as non-invasive stable and sensitive biomarkers for early diagnosis of bladder cancer, also it could be used in monitoring and prognosis of tumor at different stages.
